# Case Report: Bilateral reexpansion pulmonary edema following treatment of a unilateral hemothorax

**DOI:** 10.12688/f1000research.6000.1

**Published:** 2014-12-30

**Authors:** Steven P de Wolf, Jaap Deunk, Alexander D Cornet, Paul WG Elbers

**Affiliations:** 1 Department of Intensive Care Medicine, VU University Medical Center, De Boelelaan 1117, 1081 HZ, Amsterdam, Netherlands; 2 Department of Anesthesiology, VU University Medical Center, De Boelelaan 1117, 1081 HZ, Amsterdam, Netherlands; 3 Department of Trauma Surgery, VU University Medical Center, De Boelelaan 1117, 1081 HZ, Amsterdam, Netherlands; 4 Institute for Cardiovascular Research VU (ICaR-VU), De Boelelaan 1117, 1081 HZ, Amsterdam, Netherlands; 5 Research VUmc Intensive Care (REVIVE), De Boelelaan 1117, 1081 HZ, Amsterdam, Netherlands

**Keywords:** Re-expansion pulmonary edema, bilateral, hemothorax, pneumothorax, trauma

## Abstract

Bilateral re-expansion pulmonary edema (RPE) is an extremely rare entity. We report the unique case of bilateral RPE following a traumatic, unilateral hemopneumothorax in a young healthy male. Bilateral RPE occurred only one hour after drainage of a unilateral hemopneumothorax. The patient was treated with diuretics and supplemental oxygen. Diagnosis was confirmed by excluding other causes, using laboratory findings, chest radiography, pulmonary and cardiac ultrasound and high resolution computed tomography. His recovery was uneventful. The pathophysiology of bilateral RPE is not well known. Treatment is mainly supportive and consists of diuretics, mechanical ventilation, inotropes and steroids. In case of a pulmonary deterioration after the drainage of a traumatic pneumothorax, bilateral RPE should be considered after exclusion of more common causes of dyspnea.

## Introduction

We report here on a unique case of bilateral re-expansion pulmonary edema (RPE). First described in 1958, RPE is a rare, but well known complication of thoracocentesis ^1^. RPE usually occurs unilaterally after expansion of the ipsilateral collapsed lung caused by either spontaneous pneumothorax or various types of pleural effusion ^2^. However, in this case, RPE occurred bilaterall *y*, following expansion of a unilateral hemopneumothorax in the setting of trauma.

## Case

A 31-year old caucasian male with no significant past medical history was brought to our emergency department after falling 1.5 meters down from a platform. He was fully conscious and both respiratory and hemodynamically stable. Secondary survey findings included a fractured left olecranon and fractures of costae 7 to 9 on the left side, without clinical or radiological signs of a pneumothorax.

**Figure 1.**
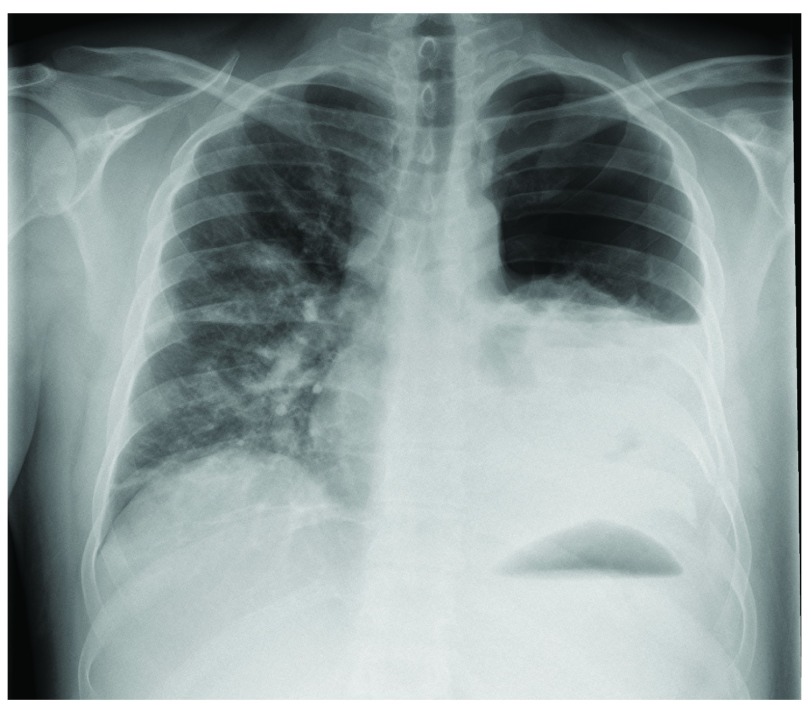
Chest radiograph showing a hemopneumothorax of the left lung.

After two days in the hospital, he underwent tension band wiring of his olecranon under general anaesthesia. There were no difficulties during mechanical ventilation. However, on the first postoperative day, his peripheral oxygen saturation was noted to be 93% without supplemental oxygen. Auscultation yielded decreased breath sounds on the left side and a chest radiograph showed a fully collapsed left lung with pleural effusion ( Figure 1). A chest tube was placed which immediately drained air and 250 mL of blood.

**Figure 2.**
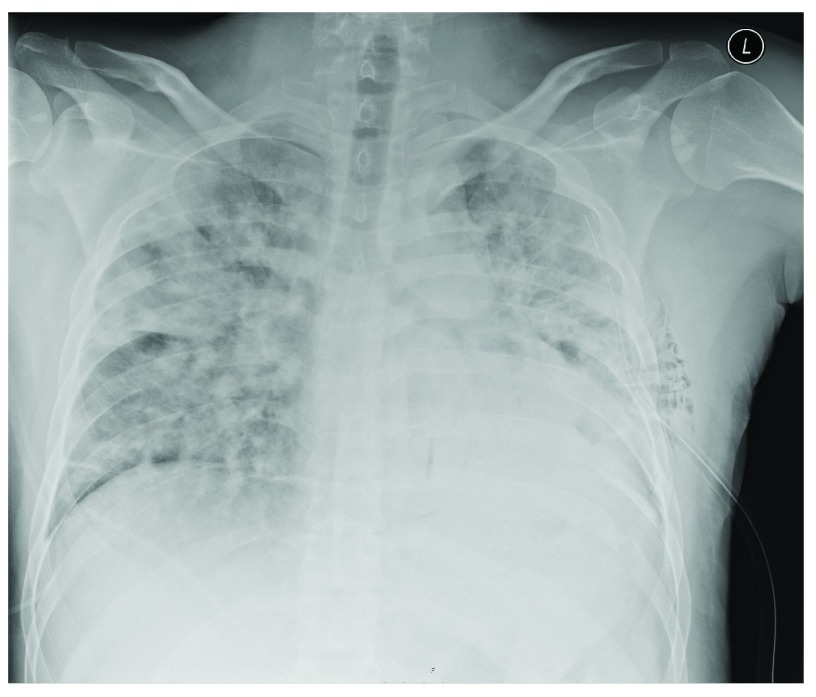
Chest radiograph one hour after drainage of the left hemopneumothorax showing bilateral pulmonary edema.

To our surprise, follow-up chest radiography one hour after drainage, demonstrated diffuse bilateral airspace opacification, peribronchial cuffing and Kerley-B lines, indicating bilateral pulmonary edema ( Figure 2). The chest tube was in a good position. In the course of several hours our patient became increasingly dyspnoeic, requiring 15 liters of oxygen via a non-rebreathing mask. He was transferred to the intensive care unit.

**Figure 3.**
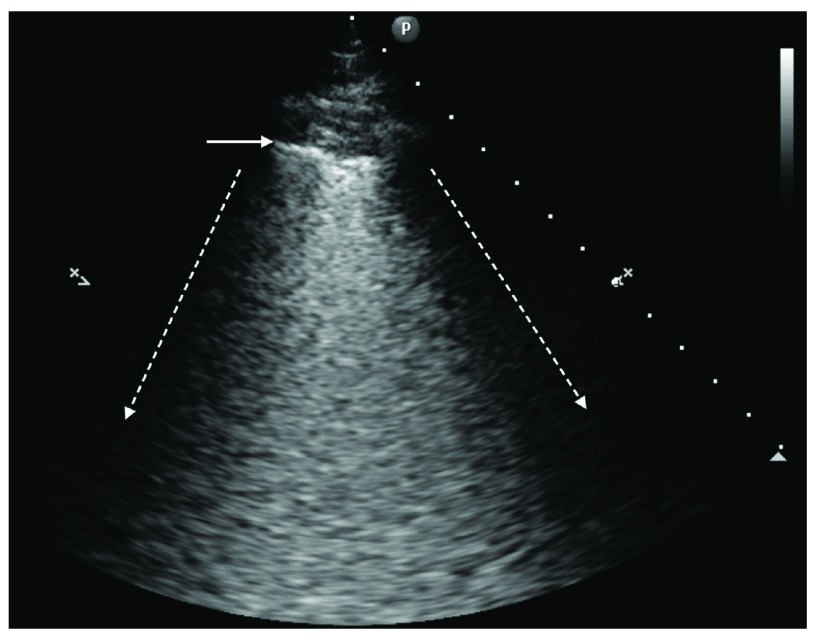
Ultrasound image of the upper right lobe. The dotted arrows indicate the rib shadows. The horizontal arrow indicates the pleura. Between the dotted arrows B-lines can be seen in a pattern called ground-glass rockets, showing an interstitial syndrome.

Intensive care ultrasound showed bilateral B-lines in all lung fields ( Figure 3), normal left and right ventricular function, no valvular dysfunction, normal atrial and caval vein dimensions and no pericardial effusion. These findings are consistent with non-cardiogenic pulmonary edema. Our patient did not receive excessive fluid therapy or blood transfusions and N-terminal-pro-B-type natriuretic peptide was normal (430ng/L), as were white cell count (9,6×10 ^9s^/L) and C-reactive protein (23mg/L). Through this process of exclusion, and consistent with recent lung re-expansion, our patient was diagnosed with bilateral RPE.

Aggressive diuretic therapy markedly improved his dyspnea without the need for mechanical ventilation and our patient was transferred back to the ward after 24 hours. Because of a persisting dependency of supplemental oxygen, high resolution computed tomography was performed two days later. This confirmed our diagnosis of bilateral pulmonary edema and revealed two additional rib fractures on the left side. Diuretics and oxygen suppletion were discontinued after a few days, and twelve days after the initial trauma our patient was discharged to home.

## Discussion

To the best of our knowledge, this is the first report of bilateral RPE following thoracocentesis of a unilateral traumatic hemopneumothorax. A few cases of bilateral RPE have been described in literature ^3– 11^. However, none of these cases were preceded by a traumatic injury. In fact, most reported cases of either unilateral or bilateral RPE followed non-traumatic pneumothorax, pleural empyema or pleural effusion. The incidence of unilateral RPE is between 0 and 6,5% whereas bilateral RPE is extremely rare ^11– 14^.

The pathophysiology of bilateral RPE is not well known. Increased levels of the pro-inflammatory cytokine interleukin-8 and monocyte chemo-attractant protein 1 might be involved in the inflammatory process that characterizes RPE ^15^. In addition, re-expansion of the lung may lead to reperfusion injury and increased permeability of the endovascular cells ^16^. A prolonged collapse seems to result in an increased risk for RPE ^4, 11^. Other risk factors include the extent of lung collapse, young age ^17^ and fast re-expansion using suction ^4^. Treatment is still mainly supportive and relies mostly on diuretics but may necessitate mechanical ventilation, inotropes and steroids ^11, 17^.

In conclusion, bilateral re-expansion pulmonary edema is an extremely rare but fascinating phenomenon following treatment of a unilateral traumatic hemopneumothorax. In case of a pulmonary deterioration after the drainage of a traumatic pneumothorax, bilateral RPE should be considered, after exclusion of more common causes of dyspnea.

## Consent

Written informed consent for publication of clinical details and clinical images was obtained from the patient.
