# Supplementary material for: Case Report: Bilateral reexpansion pulmonary edema following treatment of a unilateral hemothorax
Source: F1000Res. 2014 Dec 30;3:318. [Version 1] doi: 10.12688/f1000research.6000.1 (PMC4335596; doi:10.12688/f1000research.6000.1)
Supplement: Peer review history

# Peer review: Referee response for version 1

**DOI:** 10.5256/f1000research.6419.r7695

**Published:** 2015-02-18

**Authors:** Ehab Farag

**Affiliations:** 1 Department of General Anesthesiology and Outcomes Research, The Cleveland Clinic Foundation, Cleveland, OH, USA

I approve this case report. It is well written and informative one.

I have read this submission. I believe that I have an appropriate level of expertise to confirm that it is of an acceptable scientific standard.

# Peer review: Referee response for version 1

**DOI:** 10.5256/f1000research.6419.r7215

**Published:** 2015-01-06

**Authors:** André Coetzee

**Affiliations:** 1 Faculty of Health Sciences, Department of Anesthesiology and Critical Care, University of Stellenbosch, Tygerberg, 7505, South Africa

I approve of this submission. 

It is a rare event and the authors rather succinctly highlight the uncertainties and possible mechanisms. The post X ray is rather stunning and without the history this could indeed be confusing!

I have read this submission. I believe that I have an appropriate level of expertise to confirm that it is of an acceptable scientific standard.
